# Molecular epidemiology of sexually transmitted human papillomavirus in a self referred group of women in Ireland

**DOI:** 10.1186/1743-422X-6-112

**Published:** 2009-07-23

**Authors:** John F Menton, Suzanne M Cremin, Lydie Canier, Mary Horgan, Liam J Fanning

**Affiliations:** 1 Molecular Virology Research and Diagnostic Laboratory, Department of Medicine, Cork University Hospital & University College Cork, Cork, Ireland; 2 STI Clinic, South Infirmary Victoria University Hospital, Cork, Ireland; 3 Department of Infectious Disease, Cork University Hospital, Cork, Ireland

## Abstract

**Background:**

Human papillomavirus (HPV) causes cervical cancer and external genital warts. The purpose of this study is to document the genotype distribution of HPV in females aged between 18 and 34 who self-referred to an STI clinic with visible external genital warts (EGW). Scrapings were taken from visible external genital warts (EGW). These scrapings were analysed by PCR for the presence of HPV DNA. Positive samples were then genotyped by means of a commercially available assay (LiPA). A comparison of genotyping results determined by the LiPA assay and direct amplicon DNA sequencing was also performed.

**Results:**

Ninety-two patients out of 105 samples (88%) had detectable levels of HPV DNA. The majority of individuals with EGW (66%) showed the presence of two or more genotypes. The most common HPV genotypes present in the study population were HPV-6, HPV-11, HPV-16, HPV-18, HPV-33 and HPV-53. Potential effects of vaccination on HPV molecular epidemiology indicate that 40% of the patients could have been protected from the high risk genotypes HPV-16 and HPV-18.

**Conclusion:**

This is the first report of the molecular epidemiology of external genital warts in women aged between 18 and 34 from Ireland based on results from a LiPA assay. The study shows that most individuals are infected with multiple genotypes including those with high oncogenic potential and that the newly available HPV vaccines could have a significant impact on prevalence of the most common HPV genotypes in this study population.

## Background

Human papillomavirus (HPV) is a group of non-enveloped DNA viruses, which cause benign and malignant epithelial lesions [1]. Over a hundred different HPV genotypes have been characterized and of these more than 50% cause external genital warts (EGW) [2]. Ninety percent of EGW are reported to be of oncogenic potential, constituting genotypes 6 and 11 [3]. The majority of HPV infections are self-limiting. However, a potential long term effect of HPV infection is its association with precancerous histological lesions such as cervical intra-epithelial neoplasia 3 (CIN3) or carcinoma in situ (CIS). HPV is a necessary causative agent of cervical cancer, with HPV DNA detectable in 99.7% of cervical cancer specimens [4]. Certain types of HPV are considered high risk oncogenic such as types HPV-16, HPV-18 and HPV-31. HPV-16 alone is responsible for 58.9% of all cervical cancers [5]. Cervical cancer is the second commonest cause of cancer deaths in women worldwide. In the U.S alone the cost of preventing and treating cervical cancer exceeds $5 billion per year [6]. Cervical screening has greatly reduced the incidence of cervical cancer in the developed world [7].

An exciting advancement in cervical cancer prevention is the recent development of two HPV vaccines. A quadrivalent vaccine (Gardasil, Merck) against the four most medically significant HPV genotypes (6/11 and 16/18), which cause 90% of external genital warts and 70% of cervical cancers respectively was approved by the Food and Drug Administration (FDA) in June 2006 [8]. A bivalent vaccine (Cervarix, GlaxoSmithKline Biologicals) against HPV-16 and HPV-18 is licensed for use in Europe and it has shown potential cross protection against the high risk genotypes HPV-45 and HPV-31 [9,10]. As these vaccines become more widely used information on the molecular epidemiology of HPV in Ireland will be important to determine efficacy and the potential for cross genotype protection. It is likely that genotype displacement will occur as herd immunity increases.

The molecular epidemiology of external genital warts [EGWs] in Irish females has not been studied but likely reflects that of other European countries. The prevalence and nature of the mixed genotype infections in this population is also unknown. PCR combined with reverse line probe hybridisation (RLPH) is a fast and sensitive method of genotyping viral infections. The routine use of these assays allows a more thorough analysis of HPV molecular epidemiology and assessment of likely cervical cancer risk. The study group presented here was collated through a voluntary screening program for females, aged between 18 and 34, who self presented to an STI clinic in Southern Ireland. Scrapings from EGW were taken and tested by PCR for HPV DNA; positive samples were genotyped using a commercially available RLPH assay. The DNA sequence of the preliminary amplicon was also determined for the first 52 patients. The results of this screening programme provide information on the frequency of sexually transmitted HPV strains circulating in a sample of the Irish population.

## Results

### Detection of HPV by Real-Time PCR

To determine the presence of HPV, DNA from 114 consecutive samples was applied to a SYBR Green I assay utilising MY09-MY-11 degenerate primers. To ascertain the sensitivity of the assay a serial dilution of genomic DNA known to have integrated HPV-16 viral DNA was performed (CaSki HTC98018). The assay was capable of detecting 30 copies of HPV DNA per amplification. 92 samples were found to be positive by HPV PCR using this method. Nine of the 114 samples were blinded negative controls. Therefore, taking the blinded controls into account, 92 out of 105 samples were HPV positive.

**Figure 1:**
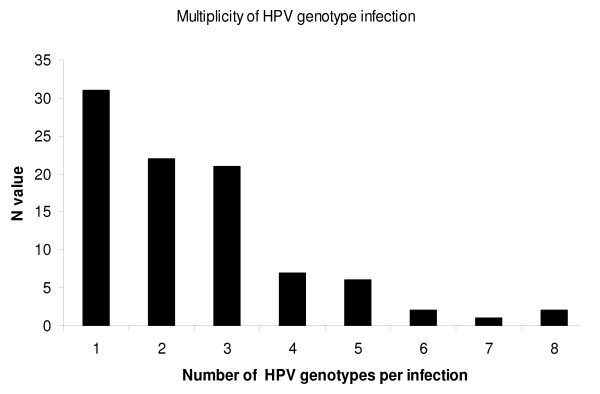
**Observed multiplicity of HPV genotype infection in external genital warts**.

In total, 21 different genotypes were recorded; 6, 11, 16, 18, 31, 33, 35, 39, 40, 45, 51, 52, 53, 55, 56, 58, 59, 66, 68, 70 and 74. These genotypes account for approximately 90% of the 17 genotypes identified as high risk or probable high risk for cervical cancer according [5]. The only two high or probable high risk genotypes not identified in this study population were genotypes 82 and 26 [5]. Thirty-one (34%) of the HPV positive patients were found to have a single HPV genotype, 97% of which were either genotype 6 or 11 (Table 1). The multiplicity of genotype infection is outlined in Figure 1. The most prevalent genotypes were HPV-16 (n = 15), HPV-18 (n = 20), HPV-31 (n = 8) and HPV-33 (n = 9) (Table 1). The maximum number of HPV genotypes detected in a single patient was 8, n = 2. Genotype 6 and genotype 11 were present in 62% and 35% of the 92 positive samples respectively (Table 1). Genotypes 6, 11 and 18 were present in the majority of dual infections. Triple infections comprised of genotype 6, 11, 16, 18 and 66 (Table 1). Other common genotypes were 39 (n = 5), 51 (n = 18), 52 (n = 8) and 53 (n = 18). The LiPA assay also confirmed that 4 patients were co-infected with both HPV-16 and HPV-18 genotypes.

**Table 1:** Profile of HPV genotypes in infected patients.

***Specimen ID***	***Genotype profile***	***Specimen ID***	***Genotype profile***
1*	6	47*	6, 18
**2**	6, 66, 40	**48**	6, 16, 53
**3**	6,11,51,53	49*	6
4*	6	50*	6
5*	11	**51**	11, 35
**6**	11, 55	**52**	11, 45
**7**	39	53*	6
**8**	6, 18, 31, 33, 51, 66, 68, 70	54*	6
**9**	6, 51, 66	**55**	6, 39, 66
**10**	11, 16, 66	56*	6
**11**	6, 11, 52, 53, 68	57*	6
**12**	6, 18, 33, 39, 52	**58**	6, 51
13*	11	**59**	11, 51, 53
**14**	11, 53	**60**	11, 18, 52, 53, 59, 70
**15**	31, 33, 53, 56	**61**	11, 16, 52, 74
**16**	6, 31, 51, 66, 68	**62**	11, 16, 51, 66
**17**	6, 59	**63**	6, 18, 31
**18**	6, 53	**64**	11, 51, 53
19*	11	**65**	6, 33, 39, 51, 53
**20**	6, 11, 66	66*	6,16,18
**21**	6, 45, 52, 53	**67**	18, 70
22*	6	68*	6
23*	6	69*	6, 11
24*	11	**70**	6, 16
25*	6, 18	71*	6
**26**	18, 53	72*	6
27*	6	73*	16
**28**	33	74*	11
**29**	6, 51, 66	**75**	18, 51, 53
30*	11	76*	11
**31**	6, 11, 40, 31, 45, 53, 70	**77**	16, 45, 59
32*	6, 16, 18	**78**	40
**33**	16, 33, 35, 51, 52	**79**	6, 18, 31, 53
34*	6	**80**	6, 16, 53
35*	11, 16	81*	6
36*	6, 18	**82**	16, 18, 58
37*	6, 18	83*	11
38*	6, 11	**84**	6, 18, 53, 68, 70
**39**	6, 11, 31, 33, 35, 39	**85**	18, 45
40*	6	**86**	6, 51
**41**	6, 31, 33, 40, 52, 53, 58, 68	87*	6
**42**	6, 11, 51	88*	6
**43**	6, 18, 45	**89**	16, 18, 51, 45
44*	6. 11	**90**	11, 18, 51
**45**	11, 51	**91**	11, 51, 52
**46**	11, 59	**92**	6, 16, 33

* indicates patients who may have been protected from a HPV infection by the quadrivalent vaccine.

### Genotyping of HPV based on Sequence analysis

52 of the 92 HPV positive samples were genotyped by direct DNA sequencing of the positive PCR product. 72% of samples were identified as having either genotypes 6 or 11; 29 samples were positive for HPV-6, 13 samples were positive for HPV-11. Only 2 samples were positive by DNA sequence analysis for HPV-16. An additional 2 samples were positive for genotype 18. Other genotypes detected were 33, 39, and 53 at a frequency of 3/52, 1/52 and 2/52, respectively.

## Discussion

The primary aim of this study was to ascertain for the first time the molecular epidemiology of sexually transmitted HPV in EGW. This genotype information should assist in estimating the potential benefits of the newly available HPV vaccines and highlight the importance of multiple infections.

DNA was successfully extracted from a limited scraping of externally visible EGW using an automated DNA extraction system. The potential falsenegative rate for this study was 12% (13/105). A possible explanation is that insufficient DNA was present to generate an amplicon or the MY09-11 primer set, used in the initial screening for HPV positivity, may be sub-optimal for specific genotypes. With regard to this latter argument, it should be noted that a total of 15 out of a possible 17 high or probably high risk genotypes were detected with this methodological approach. Of the panel of high or probably high risk genotypes proposed by Meyer *et al *only two relatively uncommon genotypes, i.e., 82 and 26, were not identified in the study population [5]. Another commonly used set of PCR primers used to amplify HPV is the PGMY09-11 primer set. Given the epidemiological coverage provided by the MY09-MY11 generic primers it is unlikely that additional significant genotype coverage would be provided by use of the PGMY09-11 primer combination [11].

The combination of the real-time and automated DNA extraction allowed for a fast sample throughput (t = 5 hours). In 100% of cases (n = 52) DNA sequencing revealed an equivalent genotype when compared to the LiPA assay. Nevertheless DNA sequencing using consensus primers doesn't readily enable the detection of multiple infections [12]. DNA sequencing with consensus primers likely reflects relative viral load with regard to the dominance of a genotype in the mixed genotype population. This feature likely affects all PCR based DNA sequencing methodologies and is not specific to the MY09-MY-11 primers used in this study. Recently, Matsukura *et al*., 2008 questioned the validity of using PCR and sequencing to determine HPV genotypes especially those associated with cervical cancer [13]. Our results would support Matsukura's inferences.

The combination of real-time PCR and LiPA assay identified low risk genotypes 6 and 11 or both in 88% of EGWs tested. This trend is evident in HPV infections worldwide as 90% of all genital warts are related to types 6 and 11 [14]. In spite of this, a clinically important detail revealed by this study was that 66% of all EGW contained multiple genotypes. Only 30/92 (30%) of the patients were infected with a low risk genotype(s). The remainder of the patients studied were infected with at least one high or probable risk HPV (Table 1).

HPV-16, HPV-18 and HPV-31 which account for 75% of cervical cancers [1] were prevalent in our study population at a combined frequency of 39% (n = 36). Although the tissue genotyped was external to the cervix, the presence of high risk genotypes in EGWs is a matter of concern and merits a thorough examination of the patient for cervical lesions and HPV genotyping of the cervix [15]. The current dogma regarding the inherent risk of HPV infection and EGW is that in 90% of patients, the HPV genotype present is associated with a low risk of progression to cervical cancer. The findings presented in this study indicate that all HPV should be genotyped by LiPA or another form of reverse line probe hybridisation, as there is a significant risk that oncogenic genotypes may be present in a co-infection scenario. The presence of oncogenic HPV in EGW raises the possibility that this infection site is a source of HPV that may infect the cervix. The correlation between the presence of multiple genotype infections in EGW and the genotype epidemiology in the epidermal tissue of the cervix is incompletely defined at present.

The optimum time for vaccination with the new HPV vaccine (subject to the duration of immune protection) is recognised to be at the onset of puberty [16]. Both the bivalent and quadrivalent vaccine could have prevented 34 patients (37%) from contracting HPV-16 and/or HPV-18. If the potential cross protection associated with the bivalent vaccine is taken into account, a further 14 patients would not have contracted either HPV 45 (n = 7) and/or HPV 31 (n = 8) (Table 1) [10]. One patient was co-infected with both genotypes 45 and 31, specimen ID 31.

## Conclusion

This study identifies for the first time the molecular epidemiology of HPV in the EGWs of young Irish women. The results of the study found that the majority of EGWs contained the low risk genotypes 6 and 11. Despite this, a large number 70% of the patients were co-infected with one or more high risk genotypes. The new HPV vaccines would have prevented a significant number of these patients from contracting HPV or provided protection against the most oncogenic HPV genotypes 16, 18 and 31. It is to be presumed that with sufficient usage of these vaccines that genotype replacement will become a feature of the epidemiology of HPV infection.

## Methods

### Ethical approval

The study outlined here was approved by the Clinical Research Ethics Committees of the Cork teaching Hospitals and University College Cork, Ireland. Participation within the study was voluntary and patients gave informed consent before entry into the study.

### Study population

Between October 2006 and October 2008 samples were taken from 114 females who self referred to an STI clinic in Cork, Ireland. The age of the patients ranged from 19–34 years of age with an average age of 24.

### Samples

Scrapings were taken from visible external genital warts of the patients using small brushes. The brushes were stored at room temperature in 500 μl of Lysis Binding buffer (LBB) from the ROCHE™ MagNa Pure LC Total Nucleic Acid isolation Kit. Samples were received from the clinician in a blinded manner. 9 random negative samples were included and the knowledge of these and individual patient status was kept hidden from the diagnostic laboratory for the duration of the investigation. Subsequent to study completion sample identities were revealed.

### Viral DNA extraction

300 μl of sample HPV-LBB was added to 200 μl of nuclease free water and applied to the MagNa Pure LC Total Nucleic acid isolation Kit using the Roche Magna Pure Robotic extraction machine, yielding 50 μl of extracted DNA. A negative control consisting of water was included with every extraction.

### HPV detection

HPV positive samples were detected by real time PCR using My09-MY-11 generic primers [17] and SYBR Green I chemistry. Briefly, 5 μl of extracted DNA was applied to a 12.5 μl of SYBR green jumpstart Taq ReadyMix™ (Sigma Aldrich), 0.5 μl each of Primer (10 μM) and 6.5 μl of nuclease free water, yielding a final volume of 25 μl. The reaction was performed on an ABI 7500 thermocycler using the following conditions, 95°C 3 min followed by 40 cycles of 95°C for 15 s, 55°C for 1 min and 72°C for 1 min 30 s with a fluorescence read step every cycle at 72°C. Positive samples were verified using a dissociation curve which gave melting peaks of the positive PCR products between 80 – 82°C. The negative control included in the DNA extraction was included in the real time PCR.

### Sensitivity of SYBR Green I PCR assay

To ascertain the sensitivity of the assay serial dilutions of commercially available genomic DNA from the CaSki cell line (HTC98018) containing 600 copies of HPV16/genome were generated. A standard curve was created where DNA from the CaSki cell line with a range of 300,000, serially diluted by a factor of 10, to a final concentration of 3 copies per PCR. The dilutions are then applied in 5 μl volumes to the Real-time assay described above to create a standard curve.

### Genotyping of positive HPV samples

Positive samples were amplified using Biotin labelled primers provided in the INNOGENTICS HPV genotyping assay (Gent, Belgium). A no template control was performed with every biotinylated PCR to ensure no cross contamination was occurring during pre-amplification stage or during the automated process. 15 μl of each biotin labelled PCR product including the negative control was then applied to a disposable line probe strip provided and placed in an AuoLipA 30 and Reverse Line Probe Hybridisation was performed at an annealing temperature of 49°C using the HCVV3 programme. Interpretation of the genotype of the positive samples was performed using the key provided with the kit. The kit can detect the following genotypes 6, 11, 16, 18, 31, 33, 40, 45, 51, 53, 54, 58, 59, 66, 68, 70 (n = 16). In cases where the CE marked LiPA assay could not resolve the exact genotype of HPV present, the sample was applied to a second LiPA assay (version 2). The HPV LiPA version 2 assay can detect the following genotypes 6, 11, 16, 18, 31/54, 33, 35, 39, 40, 42, 43, 44, 45, 51, 52, 53, 56/74, 58, 59, 66, 68/73, 70 (n = 24). The HPV LiPA Extra assay became available towards the end of the study. This assay covers all currently known high-risk HPV genotypes and probable high-risk HPV genotypes (16, 18, 26, 31, 33, 35, 39, 45, 51, 52, 53, 56, 58, 59, 66, 68, 73, 82) as well as a number of low-risk HPV genotypes (6, 11, 40, 43, 44, 54, 70) and some additional types (69, 71, 74).

All these assays carry a CE mark which means the assays meet European union stringent quality controls in terms of sensitivity and reproducibility. Where confounding genotype heterozygosity was observed the potential genotypes were excluded from the analysis for this individual. Where the CE LiPA assay could not resolve the genotype present the sample was applied to the LiPA V2 assay or the LiPA extra assay.

### DNA sequence analysis

The PCR amplicons of the first 60 positive samples were purified using the High Pure PCR product purification kit (Roche) and air dried PCR products were outsourced to MWG (Ebersberg, Germany) for DNA sequencing. Sequencing was performed using the MY-11 primer. Fifty-two samples were successfully sequenced and these were analysed by submitting the obtained DNA sequence to the BLASTn programme available at Genbank. . All sequences were submitted to the Genbank database and accession numbers were acquired for this study (EU056593–EU056644).

## Abbreviations

HPV: Human papillomavirus; EGW: external genital wart; PCR: Polymerase chain reaction; RLPH: Reverse Line Probe Hybridisation.

## Competing interests

The authors declare that they have no competing interests.

## Authors' contributions

JFM performed DNA extractions, PCR testing, genotype determination, DNA sequence analysis and manuscript preparation. SC collected samples and patient details. LC performed some PCR testing and genotype determination. MH was involved with project design and manuscript preparation. LJF was involved in project design, project management and manuscript preparation.
